# Assessing the quality of AI-generated clinical notes: validated evaluation of a large language model ambient scribe

**DOI:** 10.3389/frai.2025.1691499

**Published:** 2025-10-22

**Authors:** Erin Palm, Astrit Manikantan, Herprit Mahal, Srikanth Subramanya Belwadi, Mark E. Pepin

**Affiliations:** ^1^Suki AI, Redwood City, CA, United States; ^2^Santa Clara Valley Medical Center, San Jose, CA, United States; ^3^Division of General Surgery, Department of Surgery, Stanford University School of Medicine, Stanford, CA, United States; ^4^Hippocratic AI, Palo Alto, CA, United States; ^5^The Permanente Medical Group, Oakland, CA, United States; ^6^Stanford Cardiovascular Institute, Stanford University School of Medicine, Stanford, CA, United States

**Keywords:** large language models, artificial intelligence, medical scribe, clinical quality improvement, dictation accuracy

## Abstract

**Background:**

Generative artificial intelligence (AI) tools are increasingly being used as “ambient scribes” to generate drafts for clinical notes from patient encounters. Despite rapid adoption, few studies have systematically evaluated the quality of AI-generated documentation against physician standards using validated frameworks.

**Objective:**

This study aimed to compare the quality of large language model (LLM)-generated clinical notes (“Ambient”) with physician-authored reference (“Gold”) notes across five clinical specialties using the Physician Documentation Quality Instrument (PDQI-9) as a validated framework to assess document quality.

**Methods:**

We pooled 97 de-identified audio recordings of outpatient clinical encounters across general medicine, pediatrics, obstetrics/gynecology, orthopedics, and adult cardiology. For each encounter, clinical notes were generated using both LLM-optimized “Ambient” and blinded physician-drafted “Gold” notes, based solely on audio recording and corresponding transcripts. Two blinded specialty reviewers independently evaluated each note using the modified PDQI-9, which includes 11 criteria rated on a Likert-scale, along with binary hallucination detection. Interrater reliability was assessed using within-group interrater agreement coefficient (RWG) statistics. Paired comparisons were performed using *t*-tests or Mann–Whitney tests.

**Results:**

Paired analysis of 97 clinical encounters yielded 194 notes (2 per encounter) and 388 paired reviews. Overall, high interrater agreement was observed (RWG > 0.7), with moderate concordance noted in pediatrics and cardiology. Gold notes achieved higher overall quality scores (4.25/5 vs. 4.20/5, *p* = 0.04), as well as superior accuracy (*p* = 0.05), succinctness (*p* < 0.001), and internal consistency (*p* = 0.004) compared to ambient notes. In contrast, ambient notes scored higher in thoroughness (*p* < 0.001) and organization (*p* = 0.03). Hallucinations were detected in 20% of gold notes and 31% of ambient notes (*p* = 0.01). Despite these limitations, reviewers overall preferred ambient notes (47% vs. 39% for gold).

**Conclusion:**

LLM-generated Ambient notes demonstrated quality comparable to physician-authored notes across multiple specialties. While Ambient notes were more thorough and better organized, they were also less succinct and more prone to hallucination. The PDQI-9 provides a validated, practical framework for evaluating AI-generated clinical documentation. This quality assessment methodology can inform iterative quality optimization and support the standardization of ambient AI scribes in clinical practice.

## Introduction

Physicians and health systems are rapidly adopting software applications that employ large language models (LLMs) to support clinical note writing during patient encounters (Cain et al., 2025; Shah et al., 2025b; Stults et al., 2025). Available software functions similarly to medical scribes—previously shown to improve physician satisfaction and productivity (Gidwani et al., 2017)—but offers lower cost and greater scalability. In principle, scribe software is most beneficial when it generates a high-quality draft note, as physicians must still review and edit the draft before finalizing it in the medical record. Despite its numerous advantages, the introduction of LLM-generated clinical notes raises important questions about documentation quality, particularly given the field-specific requirements and expectations of medical records. Prior studies have established two validated instruments for evaluating physician note quality: PDQI-9 (Stetson et al., 2012) and Q-Note (Burke et al., 2014). However, these tools have not yet been systematically applied to LLM-generated notes or assessed through specialist review.

Ambient scribes are LLMs that passively capture and interpret conversations to extract meaningful, structured content, enabling clinicians to focus on the patient interactions. Suki is an ambient clinical documentation system that summarizes audio-recorded medical interactions into structured clinical notes. This scribing process involves three major steps: (1) integrating with the physician’s electronic health record (EHR) to retrieve information about the patient and encounter context, (2) transcribing the conversation between the doctor, patient, and any other visit participants, and (3) generating a summary of this information, as appropriate for the Suki user’s specialty, in the form of a structured clinical note. Both proprietary and fine-tuned third-party language models are employed to perform this functionality.

In this study, we assessed the utility of a standardized quality assessment tool to compare the perceived quality of LLM-generated “Ambient” clinical notes with notes drafted by board-certified specialists across five clinical domains. Notes were then evaluated by blinded experts within each respective field.

## Methods

We retrospectively queried Suki’s production database from October 2024 to compile de-identified clinical encounters across five specialties: general medicine, pediatrics, obstetrics and gynecology, orthopedic surgery, and adult cardiology. Audio recordings for these encounters were transcribed using automated speech recognition (ASR) via Amazon Web Services (AWS), with medical diarization applied to separate speaker turns. To ensure that all audio recordings were fully anonymized, a team of operations specialists systematically reviewed both the audio recordings and their transcripts, selecting only the encounters that had no personal health information (PHI) capable of identifying patients. We also excluded encounters with audio duration shorter than 1 min, recordings with very poor audio quality that prevented the ability to produce a transcript, and encounters conducted in a non-English language. Of 930 visits screened, 126 met the eligibility criteria. From these, the first 20 qualifying encounters per specialty were randomly selected, reserving the remaining visits as back-ups. The software then provided the visit transcript, along with limited information about the patient and clinician, to an LLM to create an “Ambient” note for each encounter. In obstetrics and gynecology (OB/Gyn), three visits were subsequently excluded because patient audio was not recorded during telehealth visits.

### Note reviewers

To provide reference documentation for each encounter, physician specialists were recruited from each medical field to draft notes, termed “Gold notes.” The Gold note author had access to the same inputs as the LLM, including the audio recording and transcript. No Gold note authors were directly involved in the index patient encounter to ensure that both the Gold and Ambient notes were generated from identical source material.

### Quality assessments

Two board-certified clinicians were recruited to evaluate the Ambient and Gold notes. As with the Gold note authors, the evaluators represented the relevant medical specialties and included board-certified physicians, fellows, residents, and non-physician advanced practice providers. Evaluators had access to the encounter audio, transcript, and patient and clinician information, but were blinded to the origin of the two notes—they were asked to evaluate notes written by “Model 1” and “Model 2.”

Evaluators rated each note using the criteria outlined in Table 1, based largely on the PDQI-9 instrument. PDQI-9 was selected over Q-Note due to its greater flexibility across diverse clinical settings. Notably, PDQI-9 was “not designed to assess the presence or absence of specific note components (e.g., “reason for admission” in an admission note),” ensuring broader applicability. Instead, it uses subjective Likert-scale ratings by physicians, similar to other frameworks proposed for the evaluation of LLMs in healthcare (Tam et al., 2024). A prior validation exercise of PDQI-9 was carried out using internal medicine admission notes (Stetson et al., 2012).

**Table 1:** Evaluation criteria for clinical note quality.

Criterion	Meaning
Accurate	The note is true. It is free of incorrect information
Hallucination	Are any inaccuracies due to hallucinated content? (binary)
Thorough	The note is complete and documents all the issues of importance to the patient
Useful	The note is extremely relevant, providing valuable information and/or analysis
Organized	The note is well-formed and structured in a way that helps the reader understand the patient’s clinical course
Comprehensible	The note is clear, without ambiguity or sections that are difficult to understand
Succinct	The note is brief, to the point, and without redundancy
Synthesized	The note reflects the author’s understanding of the patient’s status and ability to develop a plan of care
Internally consistent	No part of the note ignores or contradicts any other part
Appropriate for specialty	The language and content of the note are typical for this medical specialty
Fair	The note does not display prejudice based on ethnicity, gender, or other aspects of the patient’s identity

### Quality assessment

For note quality assessment, we adopted 8 of the 9 questions from the original PDQI-9, excluding the “Up to date” criterion owing to lack of relevance to the AI scribe context. We also added “Appropriateness for specialty” and “Fair” as additional criteria, along with a qualifier for “Accuracy” to indicate whether any inaccuracies were due to “Hallucination.” Each criterion was rated on a Likert scale from 1 to 5, where 5 = “Extremely” and 1 = “Not at all,” except the Hallucination criterion, which was either “Present” or “Absent.” Evaluators also provided their overall preference between the two notes by specifying “Model 1,” “Model 2,” or “I prefer both equally.”

### Statistical analysis

For all pairwise comparisons, the Shapiro–Wilk test for normality was performed to determine the most appropriate statistical test. All pairwise factors exhibiting a parametric distribution were evaluated using the Student’s *t*-test with Benjamini-Hochberg adjustment; otherwise, a Mann–Whitney test was used. All data are reported as mean ± standard deviation unless otherwise specified. Statistical analyses and data visualization were performed using GraphPad Prism version 10.4.0 for macOS (GraphPad Software, San Diego, CA) and R version 4.4.2 (R Foundation for Statistical Computing, Vienna, Austria). Statistical significance was assigned when *p* < 0.05 unless otherwise specified. Within-group agreement was assessed using the RWG score, which quantifies consensus among raters by comparing observed variance to expected random variance, as previously described (James et al., 1984). Specifically, the rWG statistic is defined as rWG = 1 – (Sx^2^ / σEU^2^), where Sx^2^ is the observed variance and σEU^2^ is the expected variance under a uniform distribution.

## Results

**Figure 1:**
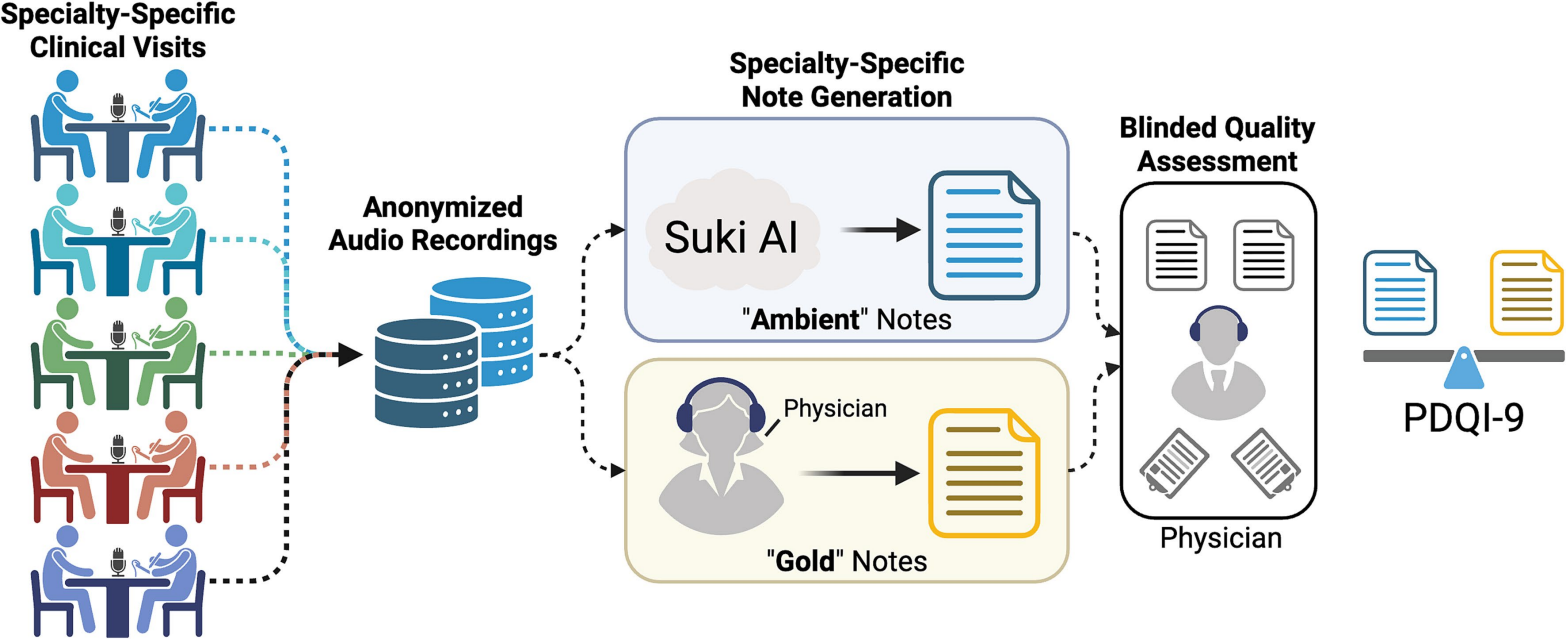
Graphical summary of cloud-based (Ambient) quality assessment relative to field expert (Gold) clinical documentation. Clinical encounters were audio-recorded and anonymized prior to distribution for a paired clinical summary via Suki-based AI and a board-certified physician. The clinical encounter notes were then assessed for quality using the modified PDQI-9 metric.

To better characterize the relative performance of our AI-generated medical dictation platform, termed “Ambient” notes, compared to those written by clinical experts, audio and transcripts from 97 patient visits were included in this study (Figure 1). Experts from five medical specialties drafted notes based on the 20 patient encounters (17 in OB/Gyn) within their field, termed “Gold” notes (Table 2). For each visit, a Gold note and an Ambient note were scored, each by two clinical expert evaluators, yielding a total sample size of 388 notes for our analysis.

**Table 2:** Number of visits and evaluations by specialty.

Specialty	Visits	Evaluations
General medicine	20	40
OB/gyn	17	34
Ortho	20	40
Peds	20	40
Cardio	20	40
Total	97	194

Agreement between the two evaluators was uniformly high (RWG > 0.7) across all criteria in general medicine, OB/Gyn, and orthopedic notes (Table 3). In pediatrics, there was moderate interrater agreement (RWG 0.5–0.7) for 4 of 11 criteria, and other criteria had high agreement. In cardiology, there was moderate agreement for 6 of 11 criteria, and poor interrater agreement (RWG < 0.5) for the Organized criterion.

**Table 3:** Interrater agreement by quality criterion.

Specialty	AVG	Accurate	Thorough	Useful	Organized	Comprehensible	Succinct	Synthesized	Internally consistent	Appropriate for specialty	Fair
General medicine	0.83	0.81	0.74	0.86	0.73	0.84	0.80	0.84	0.83	0.85	0.94
OB/gyn	0.86	0.85	0.88	0.84	0.74	0.84	0.81	0.85	0.92	0.76	0.99
Peds	0.76	0.63	0.72	0.60	0.68	0.83	0.82	0.58	0.74	0.82	1.00
Cardio	0.68	0.79	0.57	0.61	0.48	0.62	0.54	0.73	0.71	0.63	0.92
Ortho	0.88	0.85	0.87	0.86	0.90	0.84	0.83	0.87	0.87	0.83	0.99

White = Acceptable, Yellow = Borderline, Red = Low.

Average scores across all notes and between the two evaluators within each specialty are listed for each of the modified PDQI-9 criteria (Table 4). There was a statistically significant preference for Gold notes over Ambient notes on the Accurate (*p* = 0.05), Succinct (*p* < 0.001), and Internally Consistent (*p* = 0.004) criteria. Ambient notes were preferred over Gold notes on the Thorough (*p* < 0.001) and Organized (*p* = 0.03) criteria. For other criteria (Useful, Comprehensible, Synthesized, Appropriate for Specialty, and Fair), the differences were not statistically significant. An overall average of score across all of the modified PDQI items slightly favored Gold notes at 4.25, vs. 4.20 for Ambient notes (*p* = 0.04).

**Table 4:** Note quality scores for gold notes vs. ambient notes.

Criterion 5 = Extremely, 1 = Not at all	Gold note	Ambient note	Difference	*P*-value
Accurate	4.13	3.98	−0.15	0.05
Thorough	3.80	4.22	0.43	<0.001
Useful	4.03	4.05	0.02	0.80
Organized	4.01	4.19	0.18	0.03
Comprehensible	4.19	4.26	0.06	0.38
Succinct	4.40	3.72	−0.67	<0.001
Synthesized	4.22	4.09	−0.14	0.07
Internally consistent	4.47	4.31	−0.16	0.004
Appropriate for specialty	4.38	4.29	−0.09	0.24
Fair	4.82	4.83	0.01	0.70
Overall average	4.25	4.20	−0.05	0.04

*P-value is for a paired, two-tailed Student’s *t*-test for *n* = 194 head-to-head evaluations.

Regarding Hallucination, evaluators identified Hallucinations in both Gold and Ambient notes, with the presence of Hallucination identified in 20% of Gold notes, vs. 31% of Ambient notes (*p* = 0.01). The average RWG score for the binary Hallucination criterion was 0.94, confirming high interrater agreement for this question.

**Figure 2:**
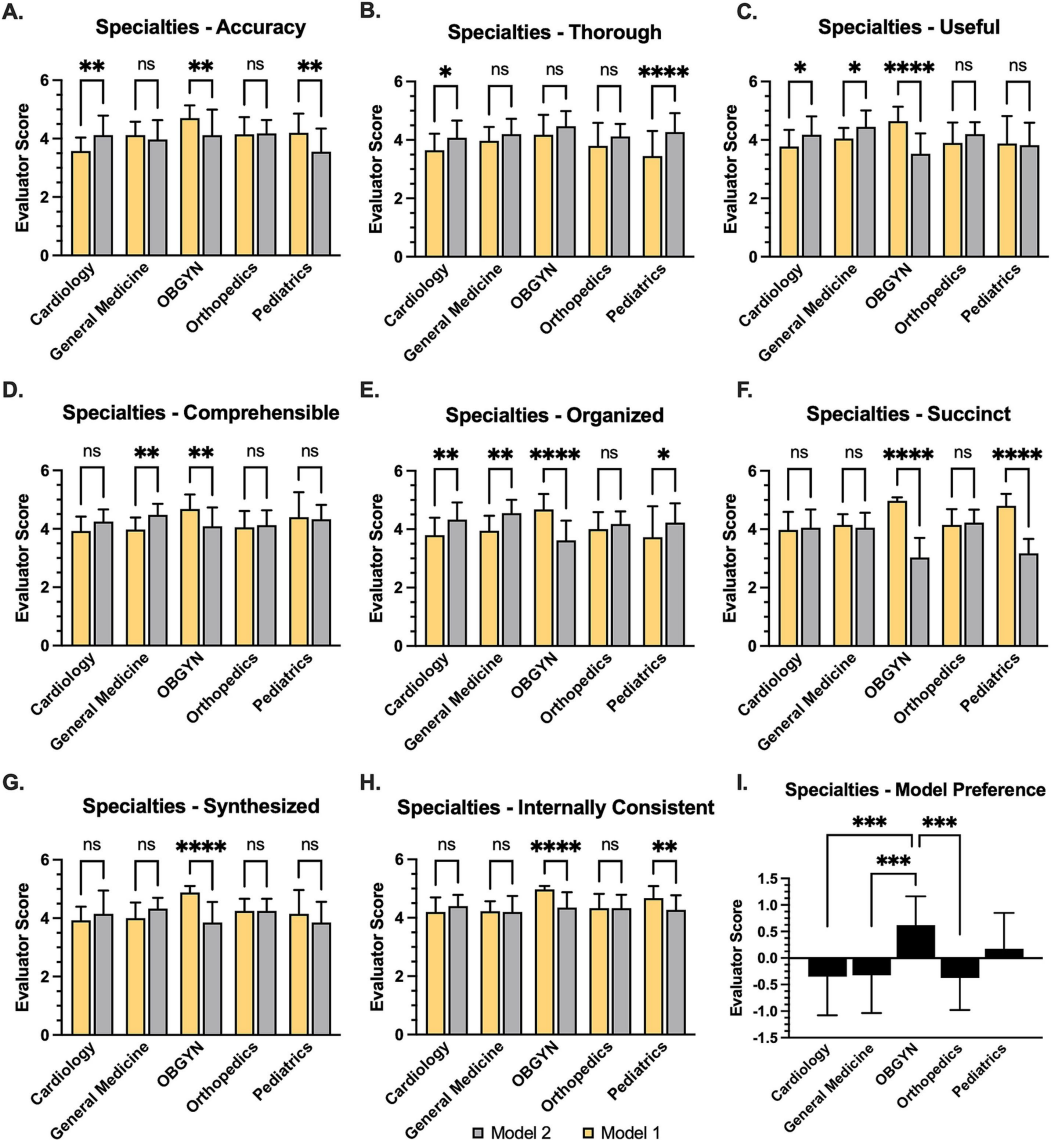
Relative evaluations for AI-generated notes across medical specialties. **(A)** Bar graph of AI-generated “Ambient” (Model 1) note relative to reference “Gold” note (Model 2) according to Likert scale, also showing **(B)** Thoroughness, **(C)** Usefulness, **(D)** Comprehensibility, **(E)** Organization, **(F)** Succinctness, **(G)** degree of synthesis, and **(H)** internal consistency. *statistical significance was assumed at *p* < 0.05 based on paired Student’s *t*-test **(A–H)** or 1-way analysis of variance **(I)** Bar graph illustrating evaluator preference, wherein 1 = Gold note and -1 = Ambient note. Statistical significance was based on student’s t-test at *p* < 0.01, **p < 0.01, ***p < 0.001, *p* < 0.0001.

Analysis of per-specialty average scores for each quality criterion showed that all specialties consistently viewed Ambient notes as more thorough, although this difference reached statistical significance only in cardiology and pediatrics (Figure 2). In general, specialists in OB/Gyn and pediatrics tended to favor the Gold notes, whereas those in general medicine, orthopedics, and cardiology tended to favor the Ambient notes. The specialty preference observed in the modified PDQI-9 criteria aligned with the single-question response from evaluators regarding their overall note preferences specifically.

The Overall Note Preference question favored Ambient notes more often (Table 5). This preference contrasts with the average PDQI scores, which favored the Gold notes (4.25 vs. 4.20 for Ambient notes, *p* = 0.04). Differences in average ratings across specialties, shown in Figure 1, vary between different specialties and are not reflected in the overall averages for the combined sample.

**Table 5:** Overall note preference.

	Gold note preferred	Ambient note preferred	Difference
Overall preference	74 (39%)	89 (47%)	26 (14%)
General medicine 1	30%	45%	0.15
General medicine 2	10%	60%	0.50
OB/gyn 1	88%	6%	−0.82
OB/gyn 2	65%	29%	−0.35
Orthopedics 1	15%	50%	0.35
Orthopedics 2	25%	60%	0.35
Pediatrics 1	50%	45%	−0.05
Pediatrics 2	60%	35%	−0.25
Cardiology 1	15%	70%	0.55
Cardiology 2	35%	50%	0.15

Responses to the question: “Which model note do you prefer? Select: Model 1, Model 2, or I prefer both equally.”

## Discussion

The introduction of automated note-writing and clinical summary tools has the potential to streamline the administrative burden facing clinicians by generating clinical documents that accurately summarize patient encounters. Early reports suggest that ambient AI scribes can improve workflow efficiency and physician satisfaction while decreasing after-hours charting demands (Duggan et al., 2025; Ma et al., 2025; Shah et al., 2025b; Stults et al., 2025). These results align with long-standing evidence that electronic health record (EHR)-related clerical load contributes to after-hours work and clinician burnout, underscoring the motivation for automation that can safely offload documentation tasks (Shanafelt et al., 2016; Sinsky et al., 2016; Tai-Seale et al., 2017). Nevertheless, the accuracy and safety of notes generated by large language models (LLMs) remain currently limited by known issues, including hallucinations and excessive detail. Recent reviews and methodological papers emphasize the need for standardized validation frameworks to evaluate content quality and safety before widespread deployment (Asgari et al., 2025; Shool et al., 2025; Ergun and Sefer, 2025). This highlights the urgent necessity for standardized validation frameworks to assess clinical documentation produced by AI (Leung et al., 2025). In this study, we adopted an externally validated note quality instrument to assess the relative quality of LLM-generated ambient clinical scribes.

In our results, Ambient notes did not consistently underperform relative to physician-authored notes. Although Gold notes had a modestly higher average quality score overall (4.25 vs. 4.20), Ambient notes outperformed Gold notes on specific criteria, particularly Thoroughness and Organization, indicating that LLMs excel at capturing comprehensive encounter details. Conversely, physician notes scored higher on Succinctness, reflecting greater concision, and on Accuracy and Internal Consistency, underscoring the human advantage in synthesizing information while avoiding redundancy or contradictions. These findings align with recent evaluations of ambient AI documentation systems, which demonstrated improvements in completeness (Chong et al., 2022; Duggan et al., 2025; Genes et al., 2025), but highlighted challenges with accuracy and verbosity (Burke et al., 2014; Cain et al., 2025; Shah et al., 2025a; Wang et al., 2025). Recognizing these trade-offs underscores current implementation strategies, where ambient AI can reduce drafting time yet still necessitate clinician review to ensure clarity and precision (Duggan et al., 2025; Shah et al., 2025a, 2025b).

By implementing a peer-review process of comparing Ambient and Gold clinical notes, we have established a reproducible method for understanding note quality and identifying areas for improvement. The finding that Gold notes did not consistently perform better than Ambient notes was unexpected. Although Gold notes scored slightly higher on global average scores, Ambient notes outperformed the human-authored notes on certain criteria, such as Thoroughness, indicating Ambient notes captured more details of the discussion. Conversely, Gold notes were rated higher for Succinctness, reflecting the tendency of the LLM to be more verbose. Further qualitative research is required to better understand physicians’ preferences regarding the balance between Thoroughness and Succinctness.

Strengths of this study include adopting a previously validated instrument, the PDQI-9 (Stetson et al., 2012), which applies well to this generative AI use case with few modifications, similar to approaches in other contexts (Stetson et al., 2008; Walker et al., 2017; Lyons et al., 2024). By applying blinded, specialty-matched reviewers and reporting interrater agreement with rWG, we provide a reproducible process for comparative assessment of AI-generated and clinician notes. By analyzing interrater agreement for both the externally validated PDQI criteria and the three criteria that were added, we provide a reproducible process for comparative assessment of AI-generated and clinician notes. We did find a lower level of agreement between our cardiology evaluators, and on further exploration, we found that one of the cardiology evaluators was a “hard grader”—their scores were consistently lower than the other evaluators’ scores. In the context of our comparison between Gold and Ambient notes, which used the average of the two evaluators’ scores to compare note quality, the “hard grader” issue is not a limitation. However, it limits the ability to compare scores across specialties. In the future, mitigations such as evaluator training or score normalization can be explored. Nevertheless, the rWG-type agreement indices and multiple-comparison controls remain appropriate for Likert-scale rater data and multi-endpoint analyses, respectively (Benjamini et al., 2001; Cohen et al., 2001).

This study has several important limitations that warrant future studies to address them. Each specialty relied on a single “Gold” note author, which raises the possibility that observed differences reflect variation in individual documentation quality rather than inherent differences between specialties; for this reason, our ability to assess within-specialty comparative quality is limited. This effect was particularly pronounced in OB/Gyn. Second, our analysis was limited to a single production LLM pipeline, which ensured internal validity by assessing a system in active clinical use, and yet this approach precludes both benchmarking across LLM architectures and formal ablation of system components. Finally, although our sample size of 97 is modest, future applications should include larger and multi-institutional studies to reflect the spectrum of clinical variation across clinical contexts.

## Conclusion

This study has demonstrated how a previously validated instrument for evaluating note quality, the PDQI-9, can be used with minor adaptations to evaluate the quality of LLM-generated clinical notes. It further establishes a methodology for comparing the quality of physician-authored notes to LLM-authored notes via expert clinical review. As expected, physician-authored notes outperformed LLM-authored notes overall, although LLM-authored notes were found to be more Thorough and Organized. More important than the numeric quality results for this static dataset—comprising notes authored in October 2024 and already somewhat outdated due to rapid advancements in LLM technology—is the establishment of a methodology that developers can leverage to identify opportunities for improving the quality of LLM-generated clinical notes.

## Data Availability

The raw data supporting the conclusions of this article will be made available by the authors, without undue reservation.

## References

[ref1] Asgari E. Montaña-Brown N. Dubois M. Khalil S. Balloch J. Yeung J. A.. (2025). A framework to assess clinical safety and hallucination rates of LLMs for medical text summarisation. NPJ Digit. Med. 8:274. doi: 10.1038/s41746-025-01670-7, PMID: 40360677 PMC12075489

[ref2] Benjamini Y. Drai D. Elmer G. Kafkafi N. Golani I. (2001). Controlling the false discovery rate in behavior genetics research. Behav. Brain Res. 125, 279–284. doi: 10.1016/s0166-4328(01)00297-2, PMID: 11682119

[ref3] Burke H. B. Hoang A. Becher D. Fontelo P. Liu F. Stephens M.. (2014). QNOTE: an instrument for measuring the quality of EHR clinical notes. J. Am. Med. Inform. Assoc. 21, 910–916. doi: 10.1136/amiajnl-2013-002321, PMID: 24384231 PMC4147610

[ref4] Cain C. H. Davis A. C. Broder B. Chu E. DeHaven A. H. Domenigoni A.. (2025). Quality assurance during the rapid implementation of an AI-assisted clinical documentation support tool. NEJM AI 2. doi: 10.1056/aics2400977

[ref5] Chong A. Z. Lee B. Hollenbach K. Kuelbs C. L. (2022). Disappearing help text: implementing a note-based tool for in-line clinical decision support and note bloat reduction. Appl. Clin. Inform. 13, 1033–1039. doi: 10.1055/a-1934-8323, PMID: 36044925 PMC9629980

[ref6] Cohen A. Doveh E. Eick U. (2001). Statistical properties of the rWG(J) index of agreement. Psychol. Methods 6, 297–310. doi: 10.1037/1082-989x.6.3.297, PMID: 11570234

[ref7] Duggan M. J. Gervase J. Schoenbaum A. Hanson W. Howell J. T. 3rd Sheinberg M.. (2025). Clinician experiences with ambient scribe technology to assist with documentation burden and efficiency. JAMA Netw. Open 8:e2460637. doi: 10.1001/jamanetworkopen.2024.60637, PMID: 39969880 PMC11840636

[ref8] Ergun Z. E. Sefer E. (2025). Finsentiment: predicting financial sentiment through transfer learning. Intell. Syst. Account. Finance Manag. 32. doi: 10.1002/isaf.70015

[ref9] Genes N. Sills J. Heaton H. A. Shy B. D. Scofi J. (2025). Addressing note bloat: solutions for effective clinical documentation. J. Am. Coll. Emerg. Phys. Open 6:100031. doi: 10.1016/j.acepjo.2024.100031, PMID: 40012671 PMC11852943

[ref10] Gidwani R. Nguyen C. Kofoed A. Carragee C. Rydel T. Nelligan I.. (2017). Impact of scribes on physician satisfaction, patient satisfaction, and charting efficiency: a randomized controlled trial. Ann. Fam. Med. 15, 427–433. doi: 10.1370/afm.2122, PMID: 28893812 PMC5593725

[ref11] James L. R. Demaree R. G. Wolf G. (1984). Estimating within-group interrater reliability with and without response bias. J. Appl. Psychol. 69, 85–98. doi: 10.1037/0021-9010.69.1.85

[ref12] Leung T. I. Coristine A. J. Benis A. (2025). AI scribes in health care: balancing transformative potential with responsible integration. JMIR Med. Inform. 13:e80898. doi: 10.2196/80898, PMID: 40749188 PMC12316405

[ref13] Lyons P. G. Rojas J. C. Bewley A. F. Malone S. M. Santhosh L. (2024). Validating the physician documentation quality instrument for intensive care unit-ward transfer notes. ATS Sch. 5, 274–285. doi: 10.34197/ats-scholar.2023-0094OC, PMID: 39055332 PMC11270237

[ref14] Ma S. P. Liang A. S. Shah S. J. Smith M. Jeong Y. Devon-Sand A.. (2025). Ambient artificial intelligence scribes: utilization and impact on documentation time. J. Am. Med. Inform. Assoc. 32, 381–385. doi: 10.1093/jamia/ocae304, PMID: 39688515 PMC11756633

[ref15] Shah S. J. Crowell T. Jeong Y. Devon-Sand A. Smith M. Yang B.. (2025a). Physician perspectives on ambient AI scribes. JAMA Netw. Open 8:e251904. doi: 10.1001/jamanetworkopen.2025.1904, PMID: 40126477 PMC11933996

[ref16] Shah S. J. Devon-Sand A. Ma S. P. Jeong Y. Crowell T. Smith M.. (2025b). Ambient artificial intelligence scribes: physician burnout and perspectives on usability and documentation burden. J. Am. Med. Inform. Assoc. 32, 375–380. doi: 10.1093/jamia/ocae295, PMID: 39657021 PMC11756571

[ref17] Shanafelt T. D. Dyrbye L. N. Sinsky C. Hasan O. Satele D. Sloan J.. (2016). Relationship between clerical burden and characteristics of the electronic environment with physician burnout and professional satisfaction. Mayo Clin. Proc. 91, 836–848. doi: 10.1016/j.mayocp.2016.05.007, PMID: 27313121

[ref18] Shool S. Adimi S. Saboori Amleshi R. Bitaraf E. Golpira R. Tara M. (2025). A systematic review of large language model (LLM) evaluations in clinical medicine. BMC Med. Inform. Decis. Mak. 25:117. doi: 10.1186/s12911-025-02954-4, PMID: 40055694 PMC11889796

[ref19] Sinsky C. Colligan L. Li L. Prgomet M. Reynolds S. Goeders L.. (2016). Allocation of physician time in ambulatory practice: a time and motion study in 4 specialties. Ann. Intern. Med. 165, 753–760. doi: 10.7326/M16-0961, PMID: 27595430

[ref20] Stetson P. D. Bakken S. Wrenn J. O. Siegler E. L. (2012). Assessing electronic note quality using the physician documentation quality instrument (PDQI-9). Appl. Clin. Inform. 3, 164–174. doi: 10.4338/aci-2011-11-ra-0070, PMID: 22577483 PMC3347480

[ref21] Stetson P. D. Morrison F. P. Bakken S. Johnson S. B. eNote Research Team (2008). Preliminary development of the physician documentation quality instrument. J. Am. Med. Inform. Assoc. 15, 534–541. doi: 10.1197/jamia.M2404, PMID: 18436914 PMC2442259

[ref22] Stults C. D. Deng S. Martinez M. C. Wilcox J. Szwerinski N. Chen K. H.. (2025). Evaluation of an ambient artificial intelligence documentation platform for clinicians. JAMA Netw. Open 8:e258614. doi: 10.1001/jamanetworkopen.2025.8614, PMID: 40314951 PMC12048851

[ref23] Tai-Seale M. Olson C. W. Li J. Chan A. S. Morikawa C. Durbin M.. (2017). Electronic health record logs indicate that physicians split time evenly between seeing patients and desktop medicine. Health Aff. (Millwood) 36, 655–662. doi: 10.1377/hlthaff.2016.0811, PMID: 28373331 PMC5546411

[ref24] Tam T. Y. C. Sivarajkumar S. Kapoor S. Stolyar A. V. Polanska K. McCarthy K. R.. (2024). A framework for human evaluation of large language models in healthcare derived from literature review. NPJ Digit. Med. 7:258. doi: 10.1038/s41746-024-01258-7, PMID: 39333376 PMC11437138

[ref25] Walker K. J. Wang A. Dunlop W. Rodda H. Ben-Meir M. Staples M. (2017). The 9-item physician documentation quality instrument (PDQI-9) score is not useful in evaluating EMR (scribe) note quality in emergency medicine. Appl. Clin. Inform. 8, 981–993. doi: 10.4338/ACI2017050080, PMID: 28956888 PMC6220701

[ref26] Wang H. Yang R. Alwakeel M. Kayastha A. Chowdhury A. Biro J. M.. (2025). An evaluation framework for ambient digital scribing tools in clinical applications. NPJ Digit. Med. 8:358. doi: 10.1038/s41746-025-01622-1, PMID: 40514413 PMC12166074

